# Late Researches on the Atmosphere

**Published:** 1859-10

**Authors:** 


### *Late Researches on the Atmosphere.*

(From the *New Orleans Medical and Surgical Journal.*)

The activity of chemistry, in its examinations of the accidental and essential constituents of the atmosphere, has been very great during the past year. We propose throwing together some gleanings from French and English journals on the subject, that will be of interest in a hygienic point of view.

*Iodine in the Atmosphere.*—Bouis has detected iodine in the rain-water of Paris during the months of April, May, June, July and August. Since it is the opinion of some medical men that the iodine contained in the air is not without some influence on public health, Bouis considered it a matter of importance to determine in what condition it existed in the atmosphere. The solution of this question is a very complicated matter, and he has made many fruitless experiments; but the results are so interesting that they are worth publication.

Being of the opinion, advanced by Chatin, that iodine is in a free state in the air, he endeavored to detect this substance, in the first products, in the distillation of rain-water; but he discovered that it remained in the residuum of the distillation, and, as rain-water always contains ammonia, he supposed that the iodine would be found in it as iodide of ammonia—a compound only slightly volatile. But the use of perchloride of iron showed that the iodine, in a large number of cases, was associated with organic substances that concealed its presence. If rain-water contained iodine in a free state, distillation alone would eliminate it; or if the iodine was in the condition of an iodide, the addition of perchloride of iron would make its elimination easy—but this is rarely the case.

In treating rain-water with perchloride of iron very slightly acid, brownish flakes are formed, resembling crenate of iron, in which case iodine cannot be determined in the products of the distillation; but if, after such treatment, the ochreous deposit be calcined, in the presence of carbonate of iron, the organic matter is destroyed, and then it is an easy matter to detect the iodine.

Bouis concludes that iodine is found in rain-water, sometimes in the condition of iodide of ammonium, but most frequently in combination with organic substances.

*Atmospheric Ozone.*—Dr. Moffat read a paper on the subject, at the meeting of the London Meteorological Society. He stated that a slip of paper moistened with iodide of potassium and starch becomes brown after exposure to the air, but after a longer exposure it lost this color; that if suspended over a cesspool the brown discoloration would not be produced, and if a brown slip were suspended over a cesspool it would also lose this color. "In these results," the author observes, "there are proofs of three distinct agents: one, ozone, which decomposes the iodide of potassium; the iodide being set free, produces the brown color. The second, sulphuretted hydrogen, the hydrogen of which removes the brown color by combining with the iodine, and forming hydriodic acid. The third, incompletely oxidized substances, the products of decomposition of animal and vegetable matter, which are more easily oxidable than the oxide of potassium." "As the products of putrefaction and combustion are found at the earth's surface," Dr. Moffat concludes that "the quantity of iodine must be greatest in the lowest strata of the air, and that consequently the quantity of ozone must *there* be at its minimum." He also states that where the air is stagnant ozone is at its minimum; and that as the north current is the lower stratum of air in motion, it is the minimum ozonic current, while the south current, being the higher air in motion, is the maximum.

The north current is the "death current," the south that of "sporadic diseases." The deadly effects of a calm are attributed to a concentration of the products of the decomposition of animal and vegetable substances, which substances are made innocuous by ozone, since it oxidizes them. In calms, fevers and cholera prevail, and the type depends on the degree of concentration of poison. Dr. Moffat has seen "an epidemic commencing with scarlatina run into typhus, and terminate in a disease of choleraic type, rapidly decline after cleansing and draining. We have no power over the winds, but he believes that if a south or ozoniferous current could be directed into 'fever nests,' or into cholera localities, these diseases would vanish; and in proof of the correctness of this opinion, mentions that cholera declined at Newcastle in 1853, and in London in 1854, after the setting in of the ozoniferous current."

Mr. H. S. Eaton read a paper at the same meeting of the Meteorological Society, showing, from tables, that "ozone was prevalent to the largest extent when the direction of the wind was between the south and west points of the compass, and when the amounts of rain and cloud were greatest; and that the least amount of ozone was coincident with winds having a northerly and easterly direction, and with the least amount of cloud and rain." These results, it will be observed, agree, in the main, with those of Dr. Moffat.

*Measurement of the Variable Intensity of Ozone.*—Dr. Lankester has devised an instrument for this purpose, composed of two cylinders, contained in a box, on which is wound a band of prepared paper. The arrangement is set in motion by ordinary clock-work. As the band leaves the one cylinder it is wound up by the other, and the arrangement is so managed that but a small portion of the paper is exposed at any time to the action of the air. The whole band is divided into twenty-four parts, corresponding with the hours of the day. The quantity of ozone which the atmosphere contains at different periods of the day is thus indicated by the different coloration of the divisions.—L. H. S. (*Am. Med. Monthly.*)

